# Primary malignant melanoma of the female genital tract synchronously involving the vulva and uterine cervix: A case report

**DOI:** 10.1097/MD.0000000000016366

**Published:** 2019-07-26

**Authors:** Yingxin Pang, Hang Yuan, Anji Ren, Shiqian Zhang, Peishu Liu

**Affiliations:** a Department of Obstetrics and Gynecology, Qilu Hospital of Shandong University, Jinan; b Department of Urology, Weifang Traditional Chinese Hospital, Weifang, Shandong, China.

**Keywords:** case report, cervical melanoma, vulval melanoma

## Abstract

**Rationale:**

Primary melanomas of the female genital tract are rare and usually occur in the vulva and vagina. Involvement of the cervix, uterus, and ovary are extremely rare. Surgery and adjuvant therapy remain the mainstay of treatment. The majority of patients experience poor long-term survival. This report aimed at highlighting an extremely rare case of primary melanoma of the female genitalia, synchronously involving the vulva and uterine cervix.

**Patient concerns:**

A 58-year-old multiparous female presented with postmenopausal bleeding for 10 days.

**Diagnoses:**

Speculum examination and histologic analysis of the surgical specimens revealed synchronous involvement of the vulva and uterine cervix by malignant melanoma. According to the American Joint Committee on Cancer stage grouping for melanoma, this tumor was at stage V.

**Interventions:**

The patient subsequently underwent radical surgery and postoperative chemotherapy.

**Outcomes:**

She has been on regular follow-up, and is now free of disease for 50 months after the operation.

**Lessons:**

Primary melanomas of the female genital tract have biologically aggressive characteristics. Optimal management consists of individualized surgery and adjuvant therapy. However, early recognition and prompt intervention offer maximal benefit from treatment.

## 1 Introduction

Primary malignant melanomas (MMs) of the female genital tract are rare, comprising 3% to 7% of all mucosal melanomas.^[1]^ Genital tract melanomas commonly occur in the vulva and vagina. Involvement of the cervix, uterus, and ovary is extremely rare. MM commonly presents at advanced stages. The diagnosis is confirmed by histologic examination using specific staining techniques and immunohistochemical analyses. Since more radical approaches do not improve survival, limited surgery has become the trend for optimal management.^[1,2]^ Ancillary therapeutic strategies include immunotherapy, chemotherapy, and biochemotherapy. In general, the majority of patients exhibit poor long-term survival. This report describes a rare case of primary MM of the female genital tract synchronously involving the vulva and uterine cervix.

## 2 Case presentation

**Figure 1:**
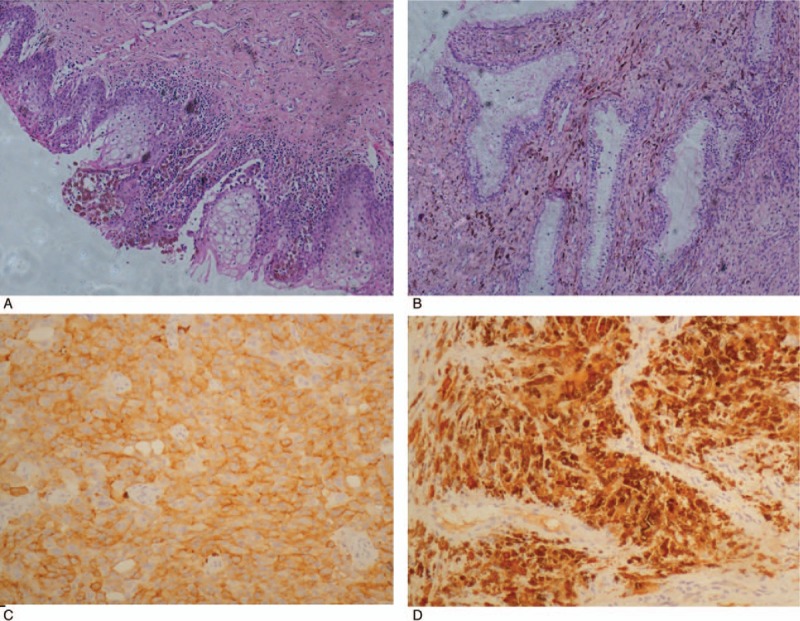
Immunohistochemistry image of (A) the vulvar malignant melanoma and (B) the cervical malignant melanoma (hematoxylin and eosin [H&E] stain; ×100). Immunohistochemistry analysis showed diffuse strong cytoplasmic reaction with (C) HMB45 and (D) S100 protein in the vulvar malignant melanoma cells (H&E stain; ×200).

A 58-year-old multiparous female visited her local hospital with a complaint of postmenopausal bleeding for 10 days. Her past and family medical histories were uneventful. She had no personal history of vaginal discharge or postcoital bleeding. In her local hospital, gynecologic examination revealed a gray-white irregular mass on the right labia minora. At the local hospital, a biopsy was taken from the mass, which measured 2.5 × 1.5 cm. Histologic examination indicated the possibility of a primary melanoma of the vulva. For further treatment, the patient was admitted to our hospital. Immunohistochemical analysis revealed that the tumor cells were strongly positive for HMB-45 and SM-100 protein, and negative for cytokeratin (Fig. 1C, D). These findings supported the diagnosis of melanoma. In our hospital, careful speculum examination showed a darkly pigmented ulcer on the right labia minora measuring about 1.5 × 1 cm in size, with a darkly pigmented mass involving the posterior lip of the cervix. The uterus was mobile and had attained a size similar to that at 50 days of pregnancy. No adnexal masses were noted, and the vagina and parametrium were not involved. An extensive assessment revealed no melanotic lesions in the eye, skin, or other mucosal sites. Additionally, computed tomography of the chest, abdomen, and pelvis showed no evidence of metastases; the tumor synchronously involved the vulva and uterine cervix.

**Figure 2:**
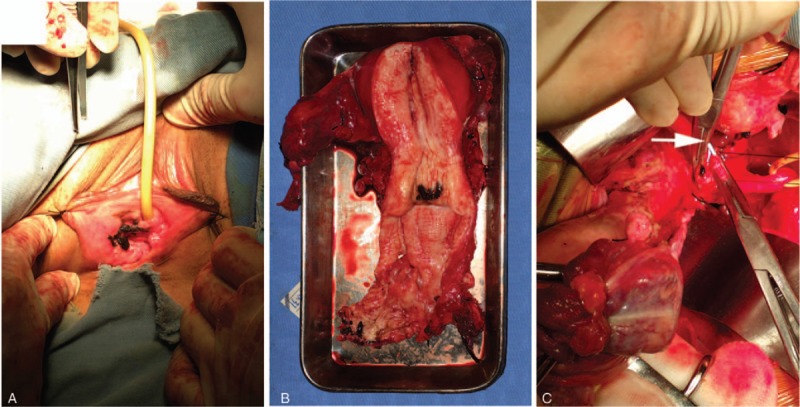
Malignant melanoma affected (A) the vulva and (B) external os of cervix and cervical canal; (C) melanin deposition in the uterine artery.

The patient subsequently underwent radical hysterectomy with bilateral salpingo-oophorectomy, total vaginectomy, partial urethrectomy, and pelvic lymphadenectomy. On gross examination, the tumor synchronously involved the vulva and uterine cervix. The depth of invasion in the vulva was 0.2 cm. The external os and cervical canal were also involved (Fig. 2A, B). During surgery, melanin deposition was also found in the uterine artery (Fig. 2C). Microscopic examination revealed a large number of tumor cells containing dark-brown intracellular pigment, both in the vulva and cervical tissues (Fig. 1A, B). The endometrium and margin of the vaginal cuff were free of tumor; none of the excised lymph nodes showed tumor metastases. According to the American Joint Committee on Cancer (AJCC) stage grouping for melanoma, this case was of stage V (T2, N0, M1). However, it was not possible to discern whether the primary site was the vulva or cervix.

The patient was started on a 4-weekly chemotherapy regimen with intravenous nedaplatin at a dose of 75 mg/m^2^ on day 1 and dacarbazine at a dose of 250 mg/m^2^ from day 1 to day 5. After 2 cycles, the dosage of the chemotherapy in the next 3 cycles was reduced owing to myelosuppression. During chemotherapy, she received 2 courses of interferon α-2b. She completed chemotherapy in July 2014. The patient has been on regular follow-up, and she is now disease free for 50 months after surgery.

## 3 Discussion

The MM is commonly considered to originating in the skin and mucosal membranes. Approximately, 3% to 7% of all melanomas occur in the female genital tract and are usually biologic aggressive. The vulva is the most common site for gynecologic melanomas, which affect women in the 5th to 7th decade of life; around 85% of cases arise in the labia minora, clitoris, or inner side of the labia majora.^[3]^ However, primary melanomas of the uterine cervix are extremely rare. Since 1950, a total of <100 cases have been reported worldwide.^[4]^ Since the uterine cervix rarely contains melanocytes, a cervical origin of melanoma has been debated. Therefore, before a diagnosis of primary cervical melanoma is made, it is of utmost importance to exclude primary melanomas at other sites. The cervix is usually involved secondarily, either by recurrent vaginal or vulval tumors or by hematogenous metastases from primaries elsewhere in the body.^[5]^ However, owing to the relatively limited blood supply and the presence of a fibrous stroma, which is not suitable for growth of a metastatic neoplasm, some controversy remains regarding metastases to the cervix.^[6]^ Interestingly, in our patient, the tumor synchronously involved the vulva and uterine cervix. This presentation has not been reported earlier in the literature. In our case, it was not possible to distinguish whether the exact primary site was the cervix or vulva. However, this distinction had no bearing on the outcome at this advanced stage. In agreement with other reports, we speculate that the uterine cervix should preferably be considered as the primary site rather than the vulva. However, in terms of prognosis, early detection of the neoplasia is more meaningful than identifying the primary site. Choriocarcinoma is the most common malignancy of the female genital tract that paradoxically metastasizes from the uterus to the vagina. Metastase from the vulva to the vagina is rarely seen in the clinic. A search of literature reveals a single report of relapse of vulvar melanoma in the cervix, 1 year after surgery.^[5]^ According to Norris and Taylor, the 4 criteria for localizing cervical melanomas are: presence of melanin in the normal cervical epithelium, absence of melanoma elsewhere in the body, demonstration of junction change in the cervix, and metastasis according to the pattern of cervical carcinoma.^[7]^ In practice, only a few cases meet these criteria.

Since this gynecologic neoplasm is relatively rare, tumor staging may be challenging. At present, the 2002 revision of the AJCC staging system for cutaneous melanomas is commonly employed for evaluating recurrence and survival in vulvar and vaginal melanomas.^[8]^ As a predictor of recurrence and survival in this disease, the International Federation of Gynecology and Obstetrics (FIGO) staging system still appears to be applicable to primary melanomas of the uterine cervix.^[1]^

Surgery is the optimal treatment of choice. For vulvar melanoma, the trend is toward more conservative surgery as there is no difference in overall survival between wide local excision and radical vulvectomy. The benefit of lymphadenectomy is also questionable. Complete regional lymphadenectomy does not seem to have any beneficial role in patients with vulvar melanoma, and should be avoided if the sentinel lymph node biopsy is negative.^[1,2]^ However, this decision showed be made with caution. For cervical melanomas, radical hysterectomy with or without pelvic lymphadenectomy is the optimal technique.^[9,10]^ In our case, we performed radical hysterectomy with bilateral salpingo-oophorectomy, total vaginectomy, partial urethrectomy, and pelvic lymphadenectomy as the melanoma involved the vulva and uterine cervix and had biologically aggressive characteristics, which was conducive to high rates of local and distant recurrence. However, it appears that the best approaches to this disease are early recognition and prompt surgical excision.

Various chemotherapy and biotherapy (i.e., immunotherapy and biologic-response modifiers) regimens have been used in locally advanced or metastatic melanomas. At present, the American Food and Drug Administration (FDA) has approved several drugs for the treatment of melanoma, which include dacarbazine and interleukin 2 for advanced melanomas, interferon α for adjuvant treatment of surgically resected high-risk disease, and ipilimumab, a monoclonal antibody against the T-lymphocyte antigen, for advanced melanomas.^[1,8]^ These drugs may be useful in the treatment of melanomas of the female reproductive tract. However, the role of chemotherapy among women with gynecologic melanomas remains unclear; in addition, the biotherapy methods described till date have been anecdotal. Reports have recommended combination chemotherapy with cisplatin, vinblastine, and bleomycin, which is possibly associated with a better response than single-agent dacarbazine.^[11,12]^ The efficacy of radiotherapy in genital melanomas remains uncertain. A few studies have shown that radiotherapy may provide benefit in patients with metastatic melanoma in the palliative setting. Despite the advanced stage, our patient responded well following surgery and combined adjuvant chemotherapy with interferon α. At present, she remains disease free for at least 50 months after surgery. On the basis of our experience, we recommend a combination of dacarbazine and platinum-based regimens in advanced gynecologic melanomas. Interferon α may be incorporated into the adjuvant regimen following standard therapy or may be administered intermittently.

In conclusion, for optimal outcomes, primary melanomas of the female genital tract should be diagnosed early with confirmation using immunohistochemistry. Although surgery remains the primary modality of treatment, adjuvant therapy should be chosen carefully. Combination regimens including chemotherapy and biotherapy may offer better survival.

## Author contributions

**Data curation:** Yingxin Pang, Hang Yuan, Anji Ren.

**Software:** Yingxin Pang.

**Investigation:** Yingxin Pang.

**Writing – original draft:** Yingxin Pang, Hang Yuan.

**Writing – review & editing:** Yingxin Pang, Hang Yuan, Shiqian Zhang, Peishu Liu.
